# Water Distribution Changes in Complex Decongestive Treatment for Leg Lymphedema: Quantitative Evaluation by Direct Segmental Multi-Frequency Bioimpedance Analysis

**DOI:** 10.3400/avd.oa.22-00037

**Published:** 2022-06-25

**Authors:** Masahiro Toshima, Yoshihisa Morino

**Affiliations:** 1 Department of Vascular Surgery, Kamiichi General Hospital, Nakaniikawa-gun, Toyama, Japan; 2 Department of Rehabilitation, Kamiichi General Hospital, Nakaniikawa-gun, Toyama, Japan

**Keywords:** segmental bioelectrical impedance analysis, extracellular water, total body water, leg lymphedema, complex decongestive treatment

## Abstract

**Background**: There is a need for a simple method for the quantitative evaluation of lymphedema swelling. In this study, we performed a direct segmental multi-frequency impedance analysis in patients with leg lymphedema.

**Methods**: The subjects were 36 patients (6 men and 30 women) with 46 lymphedema legs. The average age was 61 years. All patients had International Society of Lymphology stage II lymphedema. Swelling ratio and ultrasound subcutaneous tissue echo-free space (FS) were examined. InBody 770 was used to measure the extracellular water (ECW), intracellular water (ICW), and total body water (TBW) volumes. Changes before and after complex decongestive treatment (CDT) were examined.

**Results**: In 26 unilateral cases, the ECW, ICW, and TBW volumes of the affected legs were higher than those of the contralateral unaffected legs, and the ECW/TBW ratio was significantly higher in the affected legs (0.41) than in the contralateral unaffected legs (0.391). There was a significant correlation between the leg swelling ratio and the ECW/TBW ratio between the affected and contralateral unaffected legs (correlation coefficient=0.882). Ultrasound findings of the 46 affected legs were classified into no FS (group 0), minimal or only horizontal FS (group 1), and cobblestone-like FS (group 2). The ECW/TBW ratio of the affected legs in each group was 0.393 (14 legs), 0.407 (10 legs), and 0.426 (22 legs) respectively, demonstrating significant differences among the 3 groups. After CDT, the amount of water decreased in the affected legs and increased in the trunks and both upper limbs. The ECW/TBW ratio decreased significantly, from 0.432 to 0.414 in the affected legs, from 0.401 to 0.392 in the unaffected legs, and from 0.413 to 0.402 in the trunks. The ECW/TBW ratio had not changed and remained below 0.4 in the upper limbs.

**Conclusion**: The segmental water contents measured by direct segmental multi-frequency impedance analysis correlates well with the degree of lymphedema swelling, and subcutaneous echo findings and can demonstrate water distribution change before and after CDT, which is considered to be a useful quantitative evaluation method for lymphedema. (This is secondary publication from Jpn J Phlebol 2020; 31(1): 1–7.)

## Introduction

Volumetric measurements of lymphedema using the water displacement method, circumferential tape measurement, and infrared perometry are all employed to evaluate the swelling of lymphedema, with a volume difference of 200 mL or a circumference difference of 2 cm compared to the healthy side.^1,2)^ However, it has been pointed out that although the degree of swelling of the affected limb can be measured, the body composition of the swollen limb cannot be evaluated.^3)^

There are several methods for body component analysis, such as heavy water dilution, computed tomography, magnetic resonance imaging (MRI), dual-energy X-ray absorptiometry, and bioelectrical impedance analysis (BIA). The BIA method has been widely studied and used in the field of nutrition and fluid management in dialysis and intensive care because it is simple and non-invasive.^4,5)^

The research pertaining to BIA implementation in the diagnosis of lymphedema began to be investigated around 1990, when the amount of extracellular fluid in the affected limb was compared with that in the healthy limb. This was conducted by measuring the body’s constituent components by passing a weak electric current through the body and measuring impedance.^6,7)^ The ratio of extracellular fluid volume to intracellular fluid volume in the affected limb was then used as an index to evaluate cases of bilateral lymphedema.^8)^

However, as the existing BIA method measures whole-body impedance by considering the human body as a single cylinder, it cannot reflect the body shape, regional variations, and right–left side differences; furthermore, it cannot measure extracellular and intracellular fluid separately using only a single frequency, and the estimation formula for calculating body composition from impedance requires correction by adding statistical variables based on age and gender.^9)^

In this study, we investigated the quantitative evaluation of lower limb lymphedema using a BIA device^10,11)^ with improved development of BIA technology^12,13)^ that shows segmental water contents using direct and multi-frequency measurements.

## Subjects

Thirty-six patients with International Lymphological Association classification stage II lymphedema of the lower limbs (46 limbs in total; mean age 61 years [30–82 years]; 6 males, 30 females; 26 unilateral cases, 10 bilateral cases; 6 limbs with primary lymphedema, 40 limbs with secondary lymphedema; mean duration of disease 10 years [1–25 years]) were included.

## Methods

① Extracellular water content (ECW), intracellular water content (ICW), total body water content (TBW), and extracellular water ratio (ECW/TBW) were measured by InBody770 (InBody Co., LTD., Seoul, Korea).
② In patients with unilateral lymphedema, we measured the circumference of the lower extremity using a tape measurement, calculated the swelling ratio of the affected extremity, and compared it to the water content by BIA.
③ Subcutaneous echo free space (FS) was measured by ultrasonography and classified into three groups: absent FS (Group 0), a minimal or horizontal FS (Group 1), and cobblestone-like FS (Group 2). We compared the water content of the three groups with that of BIA.^14)^
④ We examined the changes in water content before and after complex decongestive treatment (CDT).

For statistical processing of this study, a t-test was used for comparison between the two groups, Pearson’s correlation coefficient was used for correlation between the leg swelling ratio by tape measurement and ECW/TBW, and analysis of variance table and the Games–Howell method were used for comparison of the ECW/TBW ratio of the three groups of ultrasonographic findings. A significant difference was defined as p-value <0.05.

## Results

### (I) Comparison of water content between affected and unaffected legs in 26 cases of unilateral lymphedema (**Table 1**)

The ECW and ICW of the affected leg were 2.608 L and 3.682 L, respectively, and showed a significant increase compared to ECW (1.985 L) and ICW (3.086 L) of the unaffected leg (p=0.0105, p=0.0475, respectively).

**Table 1:** Table 1 Water contents of unilateral lymphedema legs

	Unilateral lymphedema subjects (n=26)		
Water contents	Affected leg	Unaffected leg	P value
TBW.liters	6.282±2.373	5.071±1.231	0.0250
ECW.liters	2.608±1.090	1.985±0.490	0.0105
ICW.liters	3.682±1.296	3.086±0.748	0.0475
ECW/TBW ratio	0.410±0.020	0.391±0.012	0.0001

TBW: total body water; ECW: extracellular water; ICW: intracellular water; CDT: complex decongestive treatment Values are means±SD.

An ECW/TBW ratio of 0.410 of the affected leg showed a significant increase compared to 0.391 of the unaffected leg (p=0.0001).

### (II) The ECW/TBW ratio vs. the leg swelling ratio in 26 patients with unilateral lymphedema (**Fig. 1**)

The leg swelling ratio was calculated by measuring the circumference of the lower extremity at three points (thigh, lower leg, and ankle) with a tape and dividing the difference between the circumference of the affected leg and that of the unaffected leg by the circumference of the unaffected leg, and the mean value was calculated.

**Figure 1:**
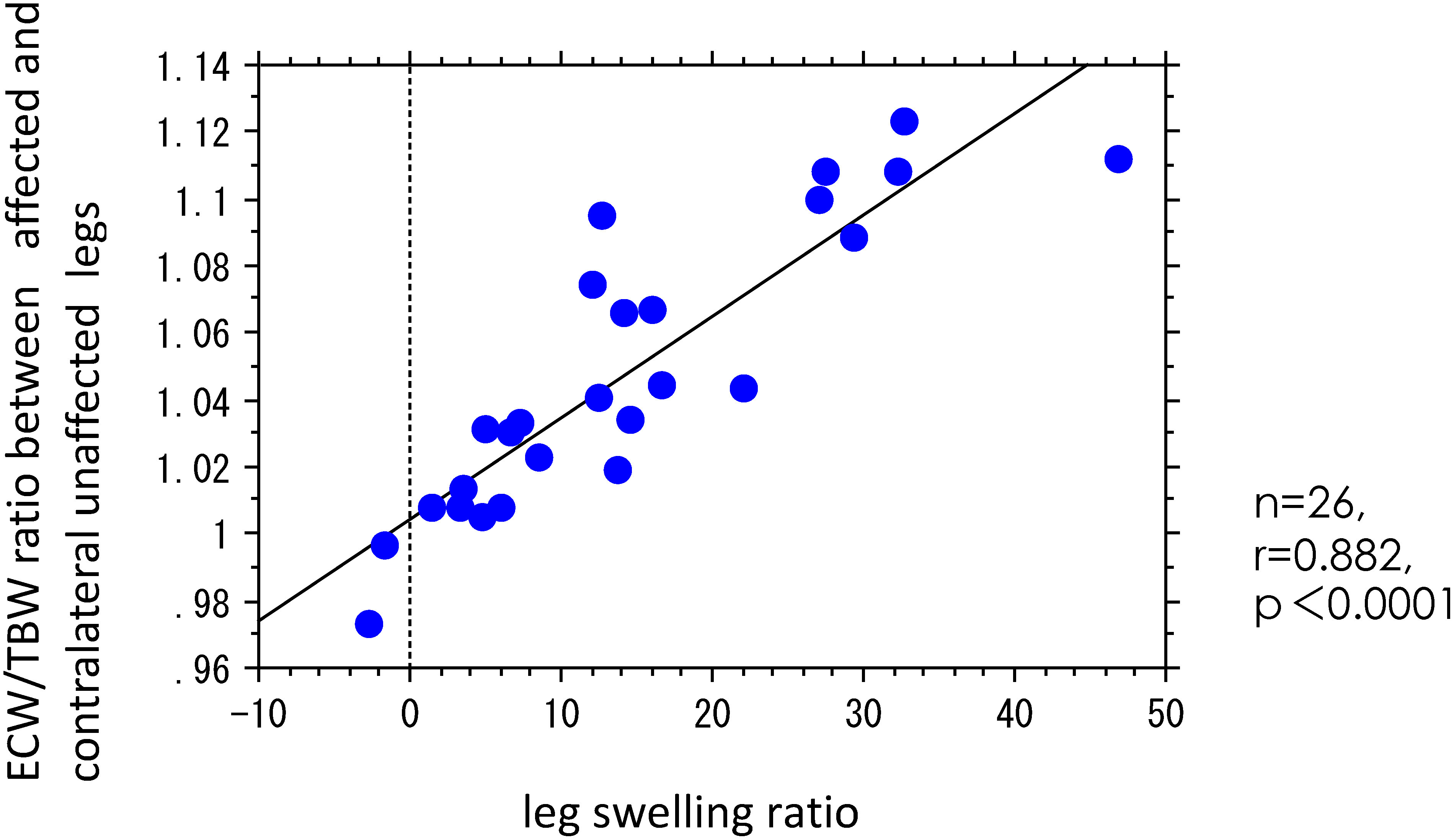
Fig. 1 Simple correlation analysis between swelling ratio and ECW/TBW ratio between aﬀected and contralateral unaﬀected legs.

ECW: extracellular water; TBW: total body water

A significant correlation coefficient of 0.882 was found between the leg swelling ratio and the ECW/TBW ratio of the affected legs to the unaffected legs (p<0.0001).

### (III) Ultrasound findings of fluids accumulation vs. the ECW/TBW ratio of the affected leg (**Fig. 2**)

Forty-six legs affected by lymphedema were examined by ultrasonography and compared to ECW/TBW ratio of the affected leg. Furthermore, 14, 10, and 22 lower extremities were classified to FS 0, 1, and 2, respectively.

**Figure 2:**
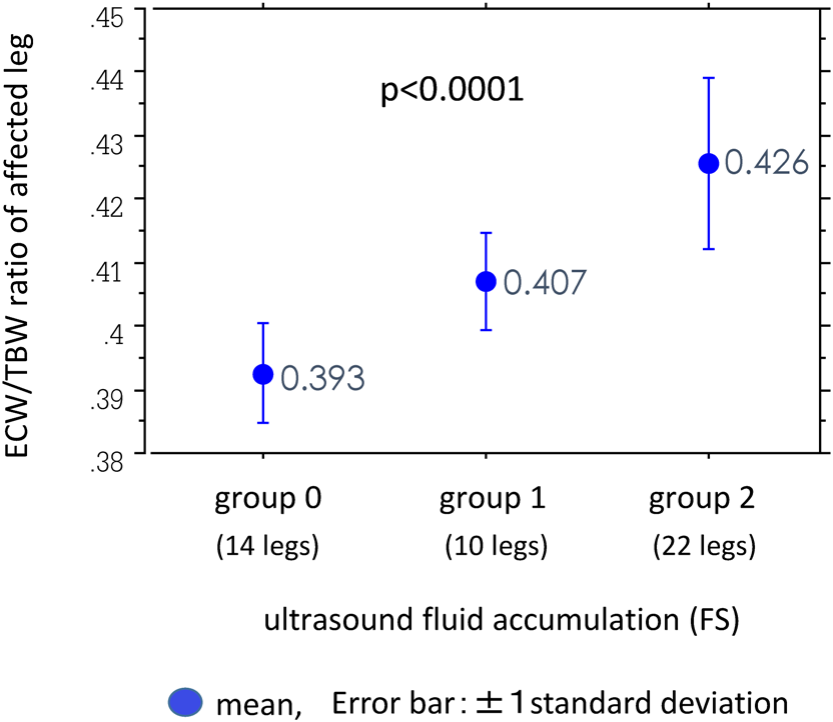
Fig. 2 Ultrasound ﬂuids accumulation and ECW/TBW of aﬀected legs.

FS: echo-free space; grade0, none; grade1, minimal or only horizontal FS; grade2, cobblestone-like FS ECW: extracellular water; TBW: total body water

Values are means.

The ECW/TBW ratio of the affected leg were 0.393, 0.407, and 0.426 in the FS 0, 1, and 2 groups, respectively, showing a significant difference among the three groups (p<0.0001).

The ECW/TBW ratio increased in proportion to the degree of fluid accumulation in the ultrasonographic findings.

### (IV) Changes in water content before and after CDT

Eight patients (affected legs: 3 bilateral, 2 right-sided, and 3 left-sided) underwent inpatient CDT for an average of 8 days (5–12 days), and the changes in water content of each segment before and after treatment were examined.

After CDT, significant decreases in TBW, ECW, and ICW were observed in the affected legs, and significant increases were observed in the trunk and both upper limbs (**Table 2**).

**Table 2:** Table 2 Change of water contents of lymphedema legs pre and post CDT

Segment	Water contents (liters)	Pre CDT	Post CDT	P value
Affected leg	TBW	7.293±2.089	4.906±0.897	0.0009
	ECW	3.159±0.971	2.157±0.617	<0.0001
	ICW	4.134±1.129	3.078±0.801	<0.0001
Contralateral Unaffected leg	TBW	5.534±1.040	4.556±0.996	0.1403
	ECW	2.218±0.443	1.996±0.476	0.0441
	ICW	3.316±0.619	3.092±0.664	0.0769
Trunk	TBW	12.600±3.138	13.812±3.165	0.0037
	ECW	5.188±1.235	5.537±1.215	0.0176
	ICW	7.412±1.924	8.275±1.972	0.0019
Rt upper limb	TBW	1.331±0.528	1.536±0.536	0.0033
	ECW	0.507±0.200	0.586±0.205	0.0045
	ICW	0.824±0.328	0.950±0.331	0.0027
Lt upper limb	TBW	1.293±0.492	1.511±0.519	0.0037
	ECW	0.496±0.189	0.576±0.198	0.0048
	ICW	0.796±0.303	0.935±0.322	0.0032

TBW: total body water; ECW: extracellular water; ICW: intracellular water; CDT: complex decongestive treatment Values are means±SD.

**Figure 3:**
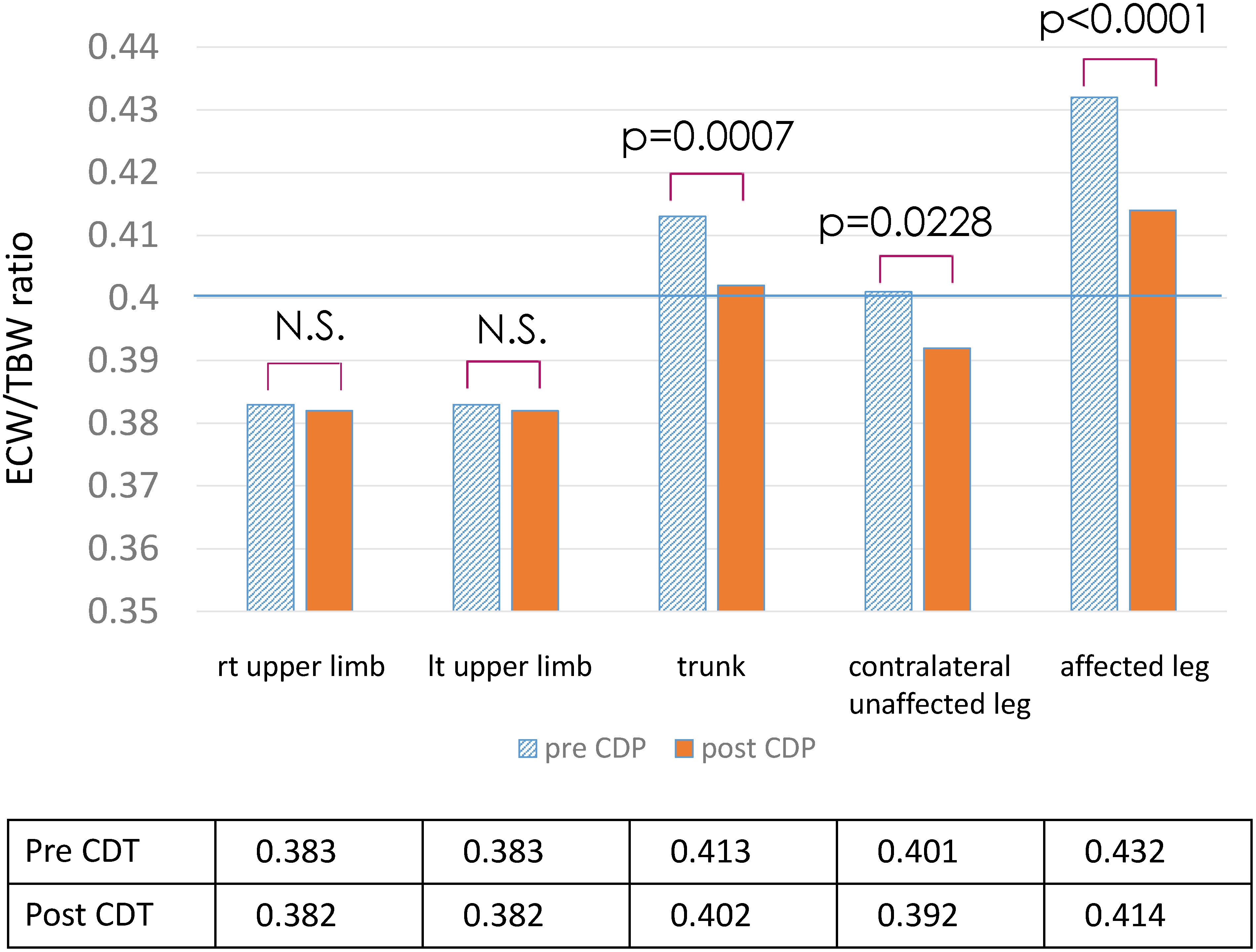
Fig. 3 Change of segmental ECW/TBW ratio pre and post CDT.

The ECW/TBW ratio of each segment showed a significant decrease from 0.432 to 0.414 (p<0.0001) in the affected leg, from 0.401 to 0.392 (p=0.0228) in the unaffected leg, and from 0.413 to 0.402 (p=0.0007) in the trunk. In both upper limbs, there was no significant change (0.383 to 0.382) (**Fig. 3**).

CDT: complex decongestive treatment

Values are means.

In the upper limbs, the ECW and ICW increased, but the ECW/TBW did not change; in the trunk, the ECW and ICW increased but ECW/TBW decreased, and in the legs, the ECW, ICW, and ECW/TBW all decreased.

The change and redistribution of water components were quantitatively observed before and after CDT, which differed by segment.

## Discussion

**Figure 4:**
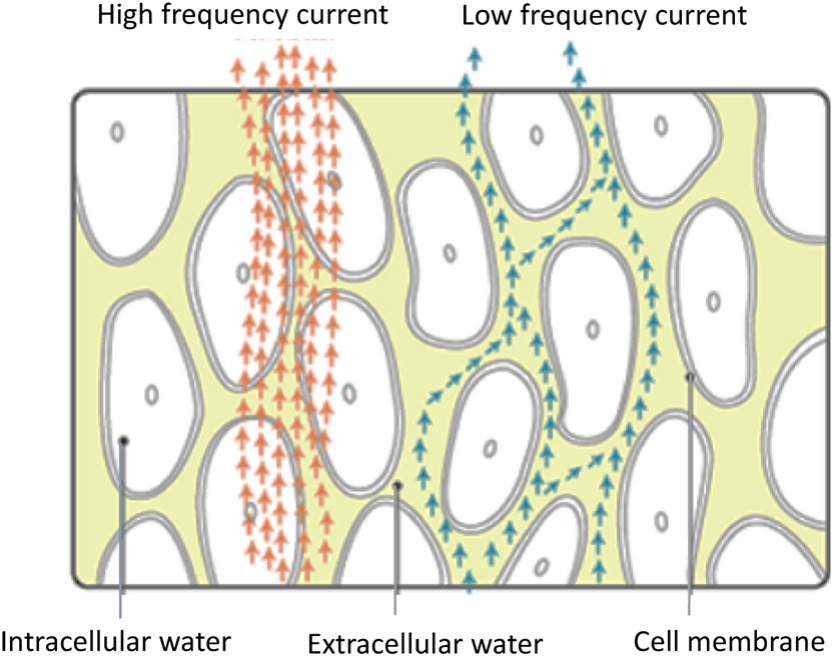
Fig. 4 Schematic diagram of tissues and current ﬂow: low versus high frequency.

In BIA, high-frequency electric current passes through the cell membrane and flows throughout the body water, whereas low-frequency electric current is difficult to pass through the cell membrane and flows along the outside of the cell membrane. Using the fact that there is a difference in the degree to which each frequency passes through the cell membrane, a multi-frequency current is applied to the body, and the amount of water inside and outside the cell is calculated separately from the ratio of each impedance obtained (**Fig. 4**).

Low frequency electrical current only passes through highly conductive extracellular ﬂuid whereas high frequency current passes through both intra- and extracellular ﬂuids, thereby measuring total ﬂuid impedance.

**Figure 5:**
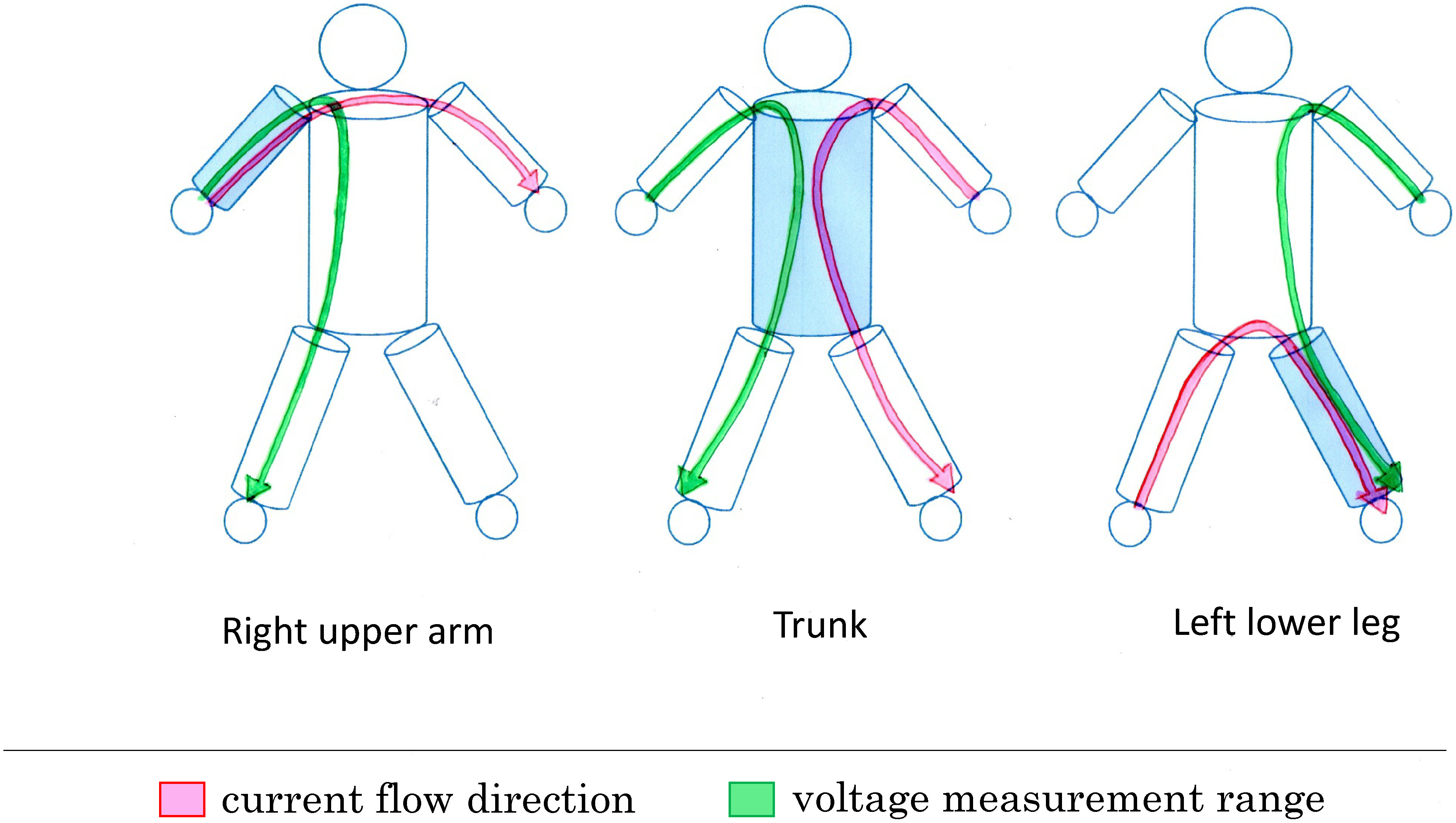
Fig. 5 Direct segmental multi-frequency BIA.

InBody770, the BIA device used in this study, a tetra-polar 8-point tactile electrode system was used. The system measured separately the impedance of participants’ right upper limb, left upper limb, trunk (from the neck down to the hip joint), right lower limb, and left lower limb at six different frequencies (1, 5, 50, 250, 500, and 1000 kHz) of current for each body segment (**Fig. 5**).

Direct segmental multi-frequency BIA was employed using the tetra-polar eight-point tactile electrode system. The system separately measured the impedance of patients’ right upper limb, left upper limb, trunk, right lower limb, left lower limb at six diﬀerent frequencies (1, 5, 50, 250, 500, 1000 kHz) for each body segment. Measurements are performed for each segment while changing the current ﬂow direction and voltage measurement range. Only the impedance where the current ﬂow and voltage measurement range overlap can be measured.

**Figure 6:**
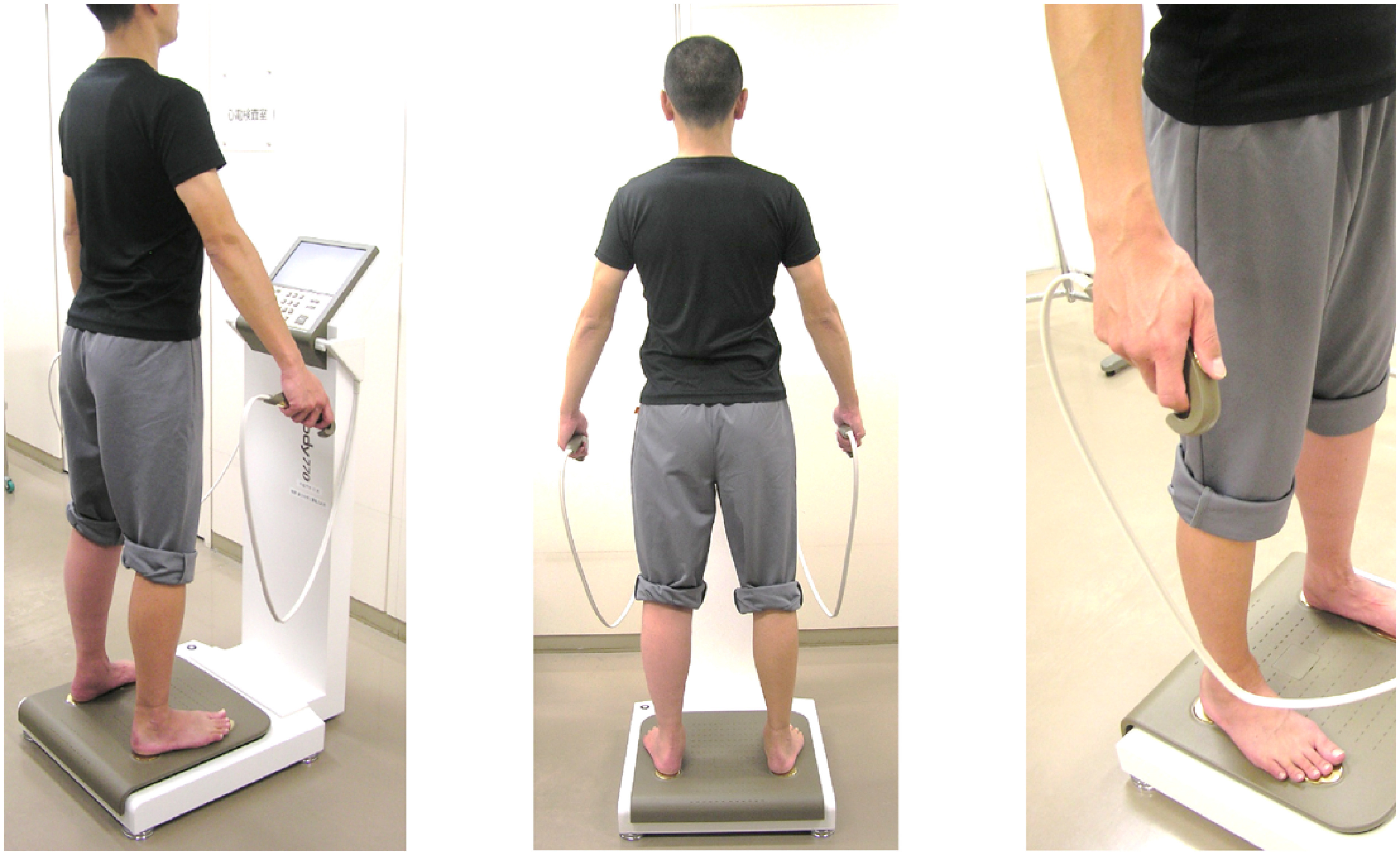
Fig. 6 Measurement with In Body770.

The measurement was performed with the patient standing barefoot on a scale with embedded electrodes, holding the handles of the analyzer in both hands, and both upper limbs abducted by approximately 30 degrees. The segmental water content was measured using an internal software formula based on height, weight, and impedance. It is a simple, non-invasive method with a measurement time of 60 seconds^15)^ (**Fig. 6**).

Subjects stood on the platform of the device barefoot with the soles of their feet on the electrodes. Subjects then grasped the handles of the unit with their thumb and ﬁngers to maintain direct contact with the electrodes. They stood for 1 min while maintaining their elbows fully extended and their shoulder joint abducted to approximately a 30-degree angle.

In this study of unilateral lymphedema of the lower extremity, both intracellular and extracellular water content increased significantly in the lymphedema leg compared to the healthy leg, with extracellular water content increasing by 31% and intracellular water content increasing by 19%. In addition, 51% of the total increase in water content was in ECW. In lymphedema of the lower extremities, the increase in extracellular water content was predominant, and the results were similar to those reported by Ward et al.,^16)^ who found in the patients of the unilateral arm lymphedema that 60% of the increase in the upper limb volume on the affected side compared to the healthy side was ECW.

In cases of lymphedema with advanced fibrosis of the subcutaneous tissue, the liquid component decreases and the fat component increases, resulting in a mixed image with lipoedema.^16)^ For lymphedema stage III and above, it is necessary to consider other evaluation methods such as skin hardness measurement.^2,17)^

This study showed that both ECW and ICW were increased by the BIA method in the lower limbs of lymphedema with International Lymphological Association Stage II, and the involvement of increased ECW was significant, thus quantitatively evaluating the pathophysiology of water composition in lymphedema.

Standard hydration status is distributed between ICW and ECW at a ratio of 62 : 38 in healthy adults.^18)^ The ECW/TBW ratio in 1992 healthy subjects aged 15–88 years was reported to be 0.360–0.397 for men and 0.372–0.403 for women using InBody770.^19)^ In clinical practice for nutritional assessment of critically ill patients, the ECW/TBW ratios between 0.36 and 0.39 are normal, between 0.39 and 0.4 indicate mild edema, and >0.4 indicate edema.^20)^

In interpreting the ECW/TBW ratio, it has been pointed out that hypoalbuminemia caused by diabetes, malignant diseases, liver cirrhosis, and poor nutritional status results in high ECW/TBW ratio due to decreased intracellular water content.^21)^ Patients of this study were cured of gynecological malignant disease and did not have any of the above factors.

In unilateral cases, coefficient of correlation between the swelling ratio according to lower limb circumference and ECW/TBW ratio on the affected side to the healthy side was 0.882, showing a high correlation. The ECW/TBW ratio of the group without subcutaneous echo FS was 0.393, and that of the group with subcutaneous echo FS was >0.4. These results suggest that the ECW/TBW value is useful for quantitative evaluation of the degree of lymphedema swelling in the lower limbs.

Combined decongestive therapy for lower limb lymphedema is widely used, and manual lymph drainage (MLD) is a part of it. In the case of MLD, pre-treatment such as shoulder exercises and abdominal breathing are performed to create a condition in which lymphatic fluid induced from the affected area can be easily absorbed, and then lymphatic drainage is practiced by massaging the affected area to move the edema fluid.^22)^

Although the benefits of MLD are recognized in clinical practice, there is little research data to conclusively support the use of MLD.^23)^ Tan et al.^24)^ reported increased lymphatic flow velocity and increased lymphatic fluid uptake with MLD in lymphedema using indocyanine green (ICG) fluorescence, and De Vrieze et al.^25)^ are in the process of testing the efficacy of MLD combined with ICG fluorescence for upper extremity lymphedema. Miranda et al.^26)^ reported on the limitations of lymphoscintigraphic evaluation, stating that although the circumference improved before and after intermittent pneumatic compression, there was no difference in the scintigraphic results, indicating that the method did not capture the movement of proteins associated with water movement. Donahue et al.^27)^ evaluated upper extremity lymphedema using MRI and quantitatively examined changes in T2 images before and after MLD by lymphedema stage, and reported differences in changes in water content composition by stage.

All of the above test methods are limited to the evaluation of local edema fluid movement, and have limitations in quantitative observation of edema fluid movement throughout the body.

For evaluation using BIA, Riegerova et al.^28)^ performed MLD on the neck and upper extremities in normal subjects using the InBody 720 and reported a decrease in water content in the entire body, a decrease in the upper extremities where massage was performed, and a mild increase in the lower extremities where massage was not performed, as well as differences in water content changes by segment.

In the present study, the CDT intervention in patients with lower extremity lymphedema resulted in a decrease in water content in the affected leg and an increase in water content in the trunk and upper extremities. In terms of the water content composition, the affected leg showed a decrease in ECW (31.7%) and ICW (25.5%), the unaffected leg showed a decrease in ECW (10.0%) and ICW (6.8%), the trunk showed an increase of ECW (6.7%) and ICW (11.6%), the right upper extremity showed an increase of ECW (15.6%) and ICW (16.1%), and the left upper extremity showed an increase of ECW (15.3%) and ICW (17.5%). In our CDT, shoulder rotation and abdominal breathing were performed on the upper extremities, abdominal and back muscle training, squatting and abdominal breathing were performed on the trunk, walking and squatting exercises were performed on the healthy lower extremities, and MLD, bandage compression, and walking and squatting exercises were performed on the affected lower extremities.

In this study, we found that the rate of water content change before and after treatment was greatest in the affected leg, which underwent the MLD plus compression therapy plus exercise, while it decreased in the unaffected leg, which was mainly exercised, and increased in the trunk, which was mainly MLD, and the upper extremity, which was lightly exercised. We could observe different water change in each segment.

We speculate that the CDT has quantitatively demonstrated that excess water in the lower limbs was transferred and redistributed to the trunk and upper limbs. The ECW/TBW ratio decreased significantly from 0.432 to 0.414 in the affected leg, from 0.401 to 0.392 in the unaffected leg, and from 0.413 to 0.402 in the trunk, while the upper limb values remained below the normal range of 0.4 with no change from 0.383 to 0.382 before and after CDT. In lymphedema, which is mainly caused by extracellular fluid retention, we believe that CDT was effective in moving edema fluid. The results of quantitative evaluation of water redistribution by this method will contribute to the evaluation of the effectiveness of CDT and its technique, and to the improvement of patients’ understanding of CDT.

## Conclusion

The BIA method showed that both ECW and ICW increased in the lower extremities of lymphedema and that the increase in ECW was largely responsible for the increase in water content, allowing us to evaluate the pathophysiology of water composition in lymphedema. In addition, the extracellular water ratio (ECW/TBW) correlated well with the degree of lymphedema swelling by the tape measurement and subcutaneous echo FS findings by ultrasonography, and the BIA method was able to display changes in water composition before and after complex decongestive therapy. The BIA method is thus useful as a quantitative assessment method for International Lymphological Association classification stage II lower limb lymphedema.

## Appendix

The content of this manuscript was presented at the 39th Annual Meeting of Japanese Society of Phlebology in July 2019 in Nagoya, Japan.

This study was approved by the Kamiichi General Hospital ethics committee.

Note from the Japanese Society of Phlebology Editorial Board: This article uses the term “complex decongestive treatment” to refer to the treatment of lymphedema. However, the term “combined therapy,” which includes lifestyle guidance and improvement in addition to “complex decongestive treatment,” is now commonly used by the Japanese Society of Lymphology and the Ministry of Health, Labor and Welfare.

## Notes

This is secondary publication from Jpn J Phlebol 2020; 31(1): 1–7.
